# The PostStroke-Manager – combining mobile, digital and sensor-based technology with personal assistance: protocol of the feasibility study

**DOI:** 10.1186/s42466-021-00137-w

**Published:** 2021-09-09

**Authors:** Dominik Michalski, Alexander Prost, Till Handel, Max Schreiber, Jean-Baptiste Tylcz, Daniela Geisler, Daniela Urban, Stephanie Schramm, Stefan Lippmann, Jenny Gullnick, Thomas Neumuth, Joseph Classen, Galina Ivanova

**Affiliations:** 1 Department of Neurology, University of Leipzig, Liebigstr. 20, 04103 Leipzig, Germany; 2 Innovation Center Computer Assisted Surgery, University of Leipzig, Semmelweisstr. 14, 04103 Leipzig, Germany; 3 Department of Primary Care, University of Leipzig, Ph.-Rosenthal-Str. 55, 04103 Leipzig, Germany

**Keywords:** Stroke, Post stroke management, Secondary prevention, Digital health, Mobile devices

## Abstract

**Introduction:**

Post stroke management has moved into the focus as it represents the only way to secure acute treatment effects in the long term. Due to individual courses, post stroke management appears rather challenging and is hindered by existing barriers between treatment sectors. As a novel concept, the PostStroke-Manager combines digital and sensor-based technology with personal assistance to enable intersectoral cooperation, best possible reduction of stroke-related disability, optimal secondary prevention, and detection of physical and psychological comorbidities.

**Methods:**

This prospective single-center observational study aims to investigate the feasibility of the PostStroke-Manager concept in an outpatient setting. Ninety patients who have suffered an ischemic or hemorrhagic stroke or transient ischemic attack will be equipped with a tablet and mobile devices recording physical activity, blood pressure, and electrocardiographic signals. Through a server-based platform, patients will be connected with the primary care physician, a stroke pilot and, if necessary, other specialists who will use web-based platforms. Via the tablet, patients will have access to an application with 10 newly designed components including, for instance, a communication tool, medication schedule, medical records platform, and psychometric screenings (e.g., depression, anxiety symptoms, quality of life, adherence, cognitive impairment). During the 1-year follow-up period, clinical visits are scheduled at three-month intervals. In the interim, communication will be secured by an appropriate tool that includes text messenger, audio, and video telephony. As the primary endpoint, feasibility will be measured by a 14-item questionnaire that addresses digital components, technical support, and personal assistance. The PostStroke-Manager will be judged feasible if at least 50% of these aspects are rated positively by at least 75% of patients. Secondary endpoints include feedback from professionals and longitudinal analyses on clinical and psychometric parameters.

**Perspective:**

This study will answer the question of whether combined digital and personal support is a feasible approach to post stroke management. Furthermore, the patient perspective gained regarding digital support may help to specify future applications. This study will also provide information regarding the potential use of remote therapies and mobile devices in situations with limited face-to-face contacts.

**Trial registration:**

German Register for Clinical Trials (DRKS00023213.), registered 27 April 2021**.**

## Introduction

Stroke treatment has traditionally focused on recanalization strategies and the establishment of specialized treatment centers [1]. These interventions have resulted in significantly reduced short-term mortality and improved functional outcome [2]. Recently, post stroke management has come into focus as stroke is increasingly understood as a chronic condition with long-lasting impairments and individual burdens [3]. Socio-economic implications arise from a recent epidemiologic projection indicating a 27% increase in stroke prevalence over the next 30 years within the European Union [4].

The complexity of post stroke management with individual courses, impairments, and needs, impedes the development of overarching concepts [5, 6]. Goals include, first, the best possible reduction of stroke-related impairments to alleviate the individual burden and enable social participation or return to work wherever possible. In this regard, barrier-free access to disease-related information and cross-sectoral communications between health care professionals could help to avoid misinformation and to tailor medical therapies to individual needs [7]. Second, the rate of secondary events, which, after cerebral ischemia, typically affect 16.8% of patients within the first 5 years [8], should be kept as low as possible. To achieve this goal, control of risk factors, e.g., by close monitoring of blood pressure and lipids, and detection of atrial fibrillation is essential, along with adequate treatment by pharmacological or lifestyle interventions [9]. Third, physical and psychological comorbidities, also occurring at later stages, should be assessed to enable early interventions. In particular, depression and anxiety symptoms were described in a relevant proportion of stroke patients [10], and are believed to impact the quality of life. In addition to these requirements contributing to a comprehensive post stroke management with a patient-centered perspective, modern strategies also include mobilizing individual skills and resources to enhance patient empowerment.

In recent years, initiatives have been launched to establish post stroke programs. Focusing on structured pathways, these programs are characterized by clinical visits with supportive elements addressing patient motivation and physical activity, risk factors and their treatment [11–14]. Unexpectedly, one such program has recently failed to show a beneficial effect regarding secondary vascular events within a follow-up period of 3.6 years [13]. However, this study has shown positive effects in secondary outcomes such as the degree of blood pressure and lipid control as also seen in another study [11]. Other programs have integrated “stroke pilots”, specially trained personnel with various supportive roles [15–18]. Final evaluations on this personal support are still pending, but practical experiences revealed signals indicating that stroke pilots are beneficial in the field of post stroke management [19].

As a first attempt to incorporate technological innovations in post stroke management, digital elements have been implemented in pilot projects as documentation tools for stroke pilots [20] or patients [15], and a communication tool between treatment sectors [21]. The beneficial effects of more complex digital interventions in other conditions [22, 23] suggest that such technology could also be a powerful tool for post stroke management [24]. Against this background, we designed the PostStroke-Manager as a novel concept combining multiple innovations based on digital and personal assistance. This concept is designed to significantly improve management after stroke with a patient-centered perspective. Using an application (app) along with mobile sensors for physical activity, blood pressure, and electrocardiographic (ECG) signals, in combination with an intensified personal assistance, this concept accompanies patients during their path after stroke. By enabling communication between disconnected healthcare sectors and free availability of individualized information to professionals, providing optimal control of risk factors independent of the patients’ location, and allowing psychometry-based screening of psychological comorbidities and impaired quality of life, such a concept would address many currently existing challenges in the post stroke management.

## Methods

### Aim of the trial

This study aims to evaluate the feasibility of the PostStroke-Manager whose concept includes a newly developed mobile, digital and sensor-based technology combined with personal assistance in post stroke management.

### Study description and study design

In a prospective single-center observational study, patients with an acute ischemic or hemorrhagic cerebral event and the ability to use mobile digital technologies, will be equipped with a commercially available tablet (Galaxy Tab S6lite, Samsung, Schwalbach, Germany) with installed PostStroke-Manager app, as well as a smartwatch with an activity tracker (TicWatch Pro 2020, Mobvoi, Hong Kong, China) and a blood pressure monitor also registering ECG signals (Veroval 2in1, Hartmann, Heidenheim, Germany). After a training phase under inpatient conditions and subsequent discharge from the hospital, patients will use the PostStroke-Manager app (see below for details) for a 1-year follow-up period, while the primary care physician, a stroke pilot, and, if necessary, specialists are connected with the patients via a web-based platform. Recruitment is planned for 1 year, beginning in fall 2021 after a brief period of technical verifications under inpatient conditions. Overall, recruitment and individual follow-up periods are expected to be completed in fall 2023 (last patient out).

### Eligibility criteria

Patients with one of the following diagnoses will be considered for participation: Ischemic or hemorrhagic stroke, defined by a persistent or transient neurological deficit in combination with an ischemic lesion or an intracerebral hemorrhage, visualized by computed tomography or magnetic resonance imaging, or transient ischemic attack (TIA), defined by a transient neurological deficit without radiological evidence of an ischemic lesion [25].

Additional inclusion criteria are: Age of at least 18 years, no or only moderate functional impairment before the qualifying event (“(pre-[modified Rankin scale (mRS)] 0-2), stable course of clinical symptoms allowing discharge from hospital, residence in the area of Leipzig/Germany, and willingness of the patients’ primary care physician to participate in the study.

The main exclusion criteria are the following: Inability to use the digital system and mobile devices (e.g., severe neglect or aphasia and hemiplegia rate as disqualifying impairments), physical impairment that could interfere with the realization of the 1-year follow-up period (e.g., cancer with a life expectancy of less than 1 year), clinically relevant psychiatric disorder, known dementia, and existing or planned pregnancy.

### Intervention

**Fig. 1:**
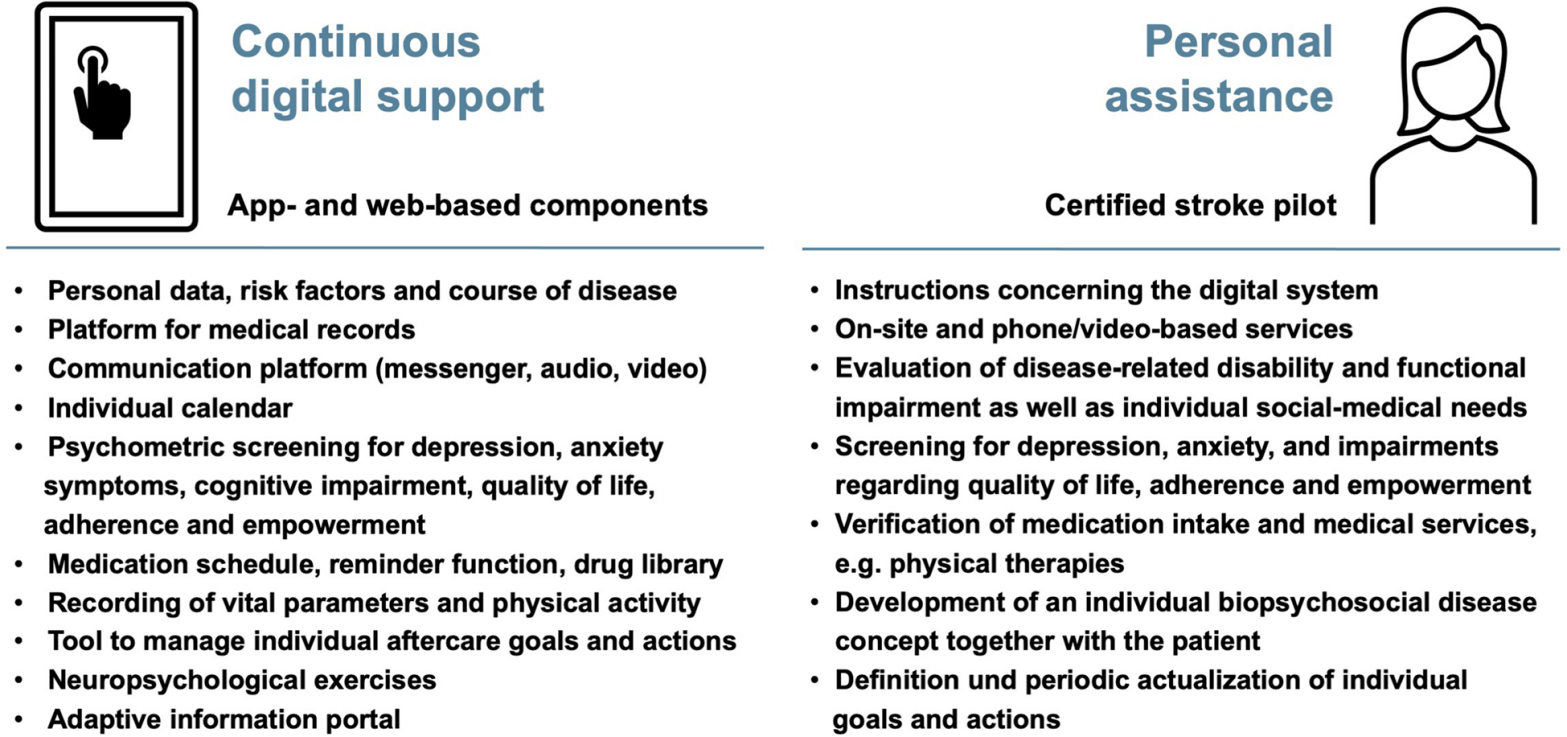
Overview of the PostStroke-Manager concept with combined digital support and personal assistance. Using a tablet with a pre-installed app, 10 components will be available for the patient that cover different aspects of post stroke management. Personal assistance will be given by a nurse with special training in case management and typical issues arising in acute and later stages of stroke. The figure including icons was created with Microsoft PowerPoint and Word for Mac (version 16.47, 2021, Microsoft, Redmond, USA)

Using the PostStroke-Manager app, patients have continuous access to 10 components (Fig. 1) designed with the following intentions: (1) to summarize information about the individual risk profile, the qualifying event, its further course (including neurological sequelae, rehabilitations and follow-up examinations), which will prevent misinformation of professionals and care-providers regarding already identified risk factors, detailed disease-related impairments and treatment plans; (2) to provide a platform for medical records of the event and upcoming medical findings, that will allow barrier-free access to essential information; (3) to enable communication between the patient, the primary care physician, and the stroke pilot as well as other specialists if necessary, via a text messenger, audio or video telephony, to reduce communication barriers for optimal treatment planning; (4) to provide a calendar function as tool for planning individual healthcare services; (5) to enable psychometric measurements on depression, anxiety symptoms, quality of life, adherence and empowerment as well as cognitive performance, which allows early detection of comorbidities; (6) to provide a medication schedule with reminder function on a daily level, which is also linked to a commercially available drug library (MMI Pharmaindex, Medizinische Medien Informations GmbH, Neu-Isenburg, Germany); (7) to record blood pressure, heart rate and ECG signals on a daily basis, ensuring optimal control of risk factors; (8) to manage individual post stroke goals and actions with respect to the individual neurological impairments and risk factors; (9) to provide neuropsychological exercises based on established tests (Bells test and trail making test) and individual recommendations (e.g., exercise sheets provided by speech therapists); and (10) to provide an information portal with, for instance, pathophysiological details of the underlying event, treatment options and details on social and financial supports from the public health system, to enhance patients’ empowerment. The digital system also includes basic technical support by a database with frequently asked questions (FAQs) and a ticket system that allows electronic feedback.

Personal assistance is provided by a stroke pilot who is an experienced nurse with special training in case management (acquired at the Dresden International University, Dresden, Germany) and additional training on stroke-related issues following the educational program of the German Stroke Foundation (Gütersloh, Germany). Specifically, the stroke pilot will help to navigate individual rehabilitation processes, which is inherently complex, particularly in the outpatient setting with varying providers of physical therapy, occupational therapy and speech therapy (Fig. 1). Furthermore, interactions with the patient are intended to evaluate individual disabilities with associated needs and goals, and also to screen for comorbidities (e.g., depression and anxiety symptoms). In addition to reviewing medication adherence and the use of physician-prescribed therapies, contacts will be used to cooperatively develop a biopsychological disease model based on individual risk factors, which plays a central role in patient empowerment. Regular visits are scheduled in 3-months-intervals with additional on-site contacts as demanded by the patient. In the meantime, support will be provided through the communication tool. To consider individual factors in terms of social and living environmental aspects (e.g., possible support by relatives, presence of an elevator in case of a higher floor level), the stroke pilot will conduct at least one home visit in the third month after study initiation. Regarding the tablet and mobile devices used by the patient, the stroke pilot provides instructions at the study entry and during the follow-up period.

Because the PostStroke-Manager app is not yet certified as a medical device, the medical aspects of individual post stroke management, such as checks of blood pressure and ECG as well as prescription of medication and physical therapies, will be provided by the primary care physician. As part of the regular care, consultations are planned in intervals of three months, which will also be used for blood-based analyses of lipids and glucose metabolism, and reassessment of current medication and needs for additional therapies.

**Fig. 2:**
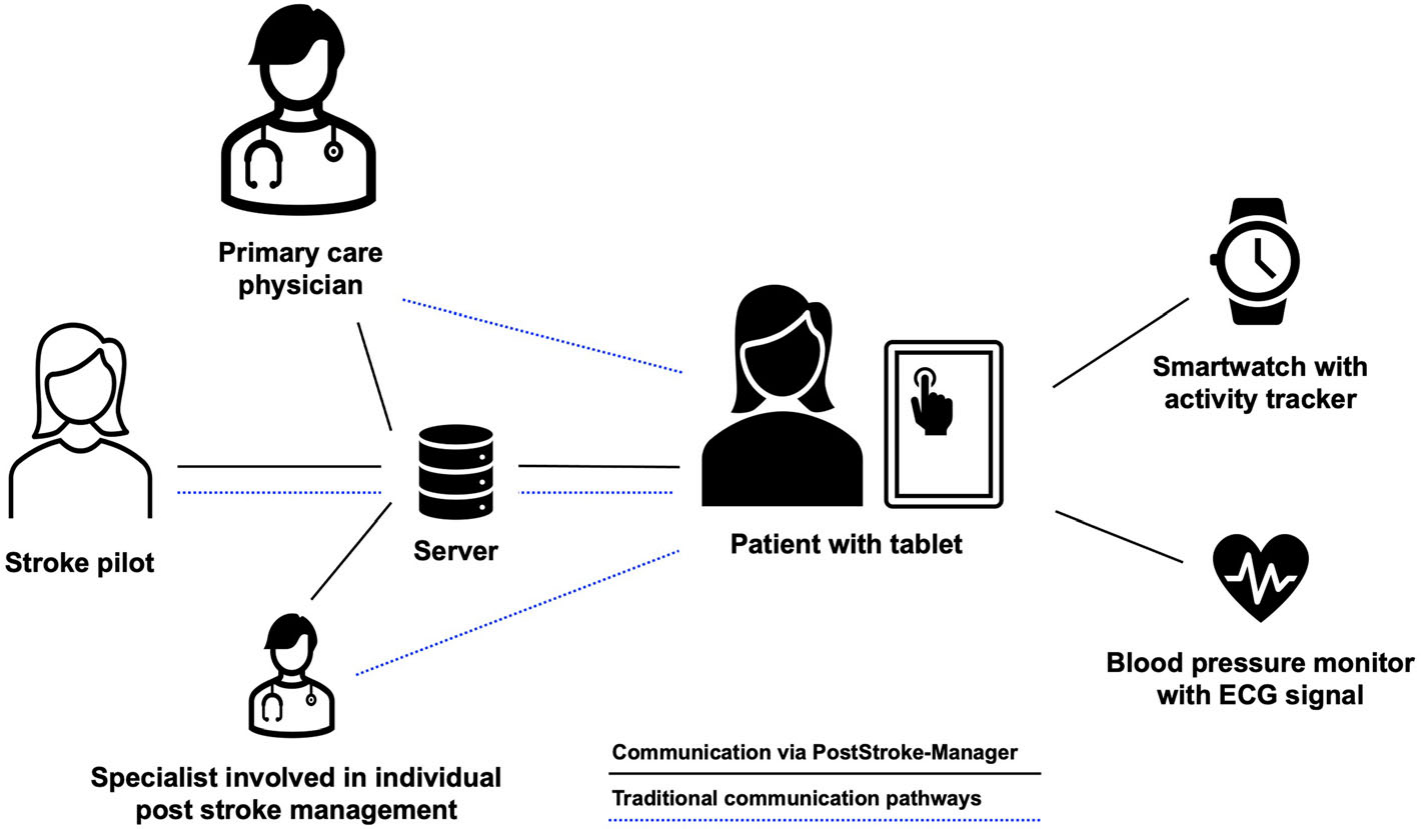
Conceptualization of the PostStroke-Manager with a central position of the patient, equipped with a tablet and mobile devices for registration of physical activity, blood pressure, and electrocardiographic signals. Using WLAN or SIM cards, the tablet will be connected to a server allowing interactions with the primary care physician, the stroke pilot as well as specialists if indicated. Thereby, the system will mimic traditional communication pathways and at the same time will add a lot of technological advances for post stroke management. Abbreviation: ECG: electrocardiography. The figure including icons was created with Microsoft PowerPoint and Word for Mac (version 16.47, 2021, Microsoft, Redmond, USA)

To enable communication between involved persons and to ensure stable registration of data obtained from the mobile devices, both the smartwatch and the blood pressure monitor are connected to a personalized tablet via Bluetooth, while the tablet is connected to a central server either via WLAN or mobile data usage (SIM card) (Fig. 2). Using web-based access to the system, stroke pilot, primary care physician and other specialists are connected to the server and have access to the individual patient data. To ensure highest levels of data protection, patients will have the central role in granting access to individual data as they voluntarily initiate connections to other persons. Thereby, connections will follow an informed consent and will technically be realized by a (QR) code.

**Fig. 3:**
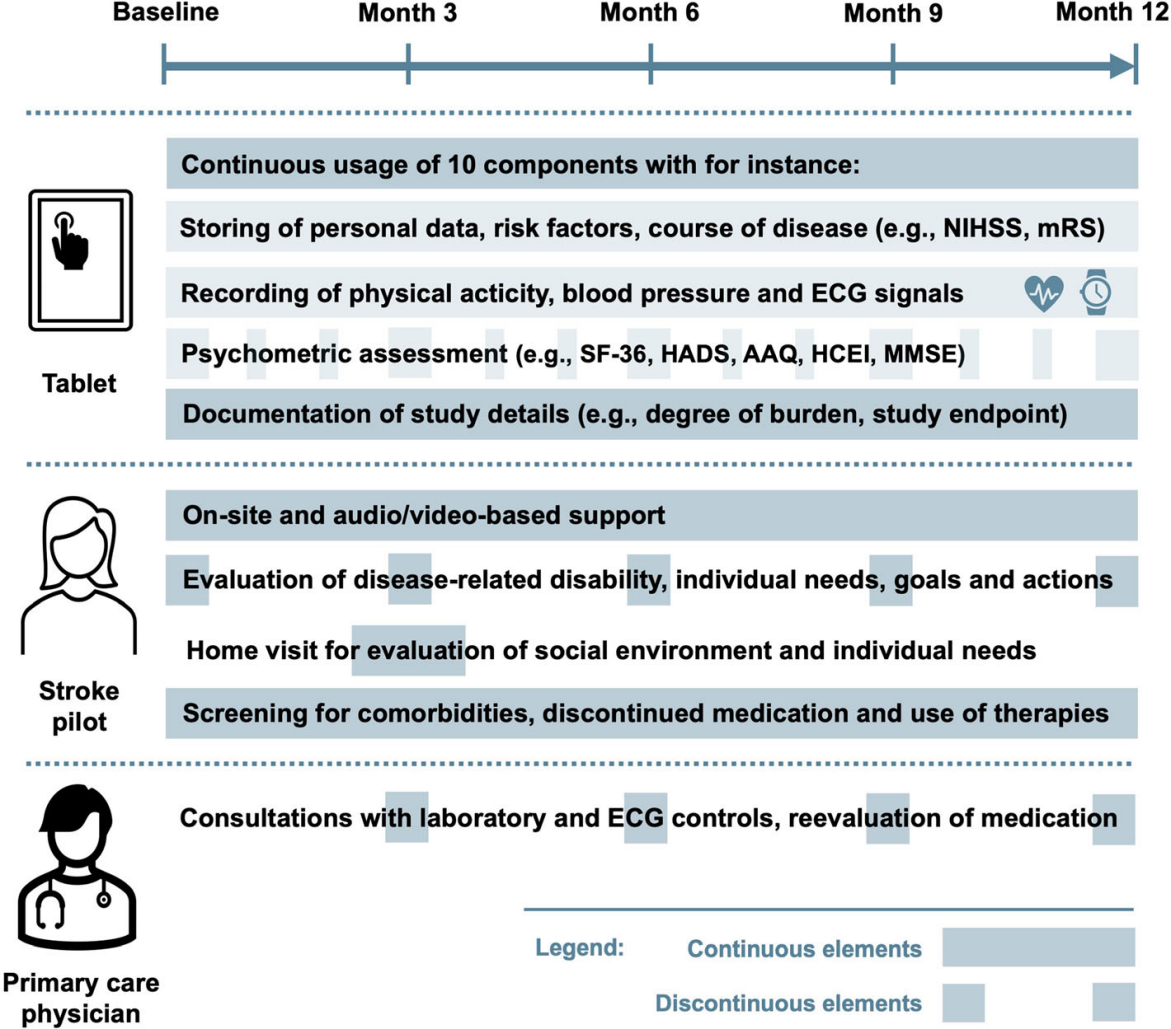
Overview of the PostStroke-Manager feasibility study. During a follow-up period of one year, visits are planned in 3-months-intervals, allowing direct interactions between the stroke pilot and the patients, while in the meantime communications will typically be arranged via the digital system. Abbreviations: NIHSS: National institutes of health stroke Scale, mRS: modified Rankin scale, SF-36: Short form (36) health survey, HADS: Hospital anxiety and depression scale, AAQ: Adherence assessment questionnaire, HCEI: Healthcare empowerment inventory, MMSE: Mini mental status examination, ECG: electrocardiography. The figure including icons was created with Microsoft PowerPoint and Word for Mac (version 16.47, 2021, Microsoft, Redmond, USA)

During the 1-year follow-up period, the combined digital-based support and personal assistance will be accompanied by quarterly arranged clinical visits performed by a study physician together with the stroke pilot (Fig. 3). Measurements for the clinical course and the psychometric testing are spaced at different intervals (Table 1). All data will be collected digitally as inputs are made entirely in the app- or web-based platform. For safety, clinical visits will also be used to check the correct usage of the mobile devices, and patients will be asked about any burden they have experienced from using the system. For this purpose, a 4-point scale was designed with the response opinions “no burden”, “low burden”, “relevant burden” and “severe burden”. In the case of a “relevant burden,” the patient will be asked to stop the study, whereas in the case of a “severe burden” participation of the patient will be terminated by study staff.

**Table 1:** Overview of clinical and psychometric measurements

Abbreviation	Full name and reference	Timepoint
*Neurological deficits and functional impairment*		
NIHSS	National Institute of Health stroke scale [26]	Baseline, months 3, 6, 9 and 12
mRS	modified Rankin scale [27]	Baseline, months 3, 6, 9 and 12
BI	Barthel index [28]	Baseline, months 3, 6, 9 and 12
*Depression and anxiety symptoms*		
PHQ-9	Patient health questionnaire 9 items [29]	Every month
HADS	Hospital anxiety and depression scale [30]	Baseline, months 3, 6, 9 and 12
*Quality of life*		
EQ-5D	EuroQoL 5 dimensions [31]	Every month
SF-36	Short form (36) health survey [32]	Baseline, months 3, 6, 9 and 12
*Adherence*		
AAQ	Adherence assessment questionnaire [33]	Baseline, months 6 and 12
ABQ	Adherence barriers questionnaire [34]	Baseline, months 6 and 12
*Empowerment*		
HCEI	Healthcare empowerment inventory [35]	Baseline, months 3, 6, 9 and 12
*Neuropsychological and cognitive impairment*		
BT	Bells test [36]	Irregular
TMT	Trail making test [37]	Irregular
MMSE	Mini mental status examination [38]	Baseline, months 3, 6, 9 and 12

In case of restrictions for face-to-face contacts that may occur in the context of the SARS-CoV-2 pandemic, critical visits are planned digitally. For this purpose, the communication platform is available for video-based interactions, while data collected with the tablet and mobile devices will generally be transferred to the server and thus become available without physical contacts.

### Outcome measures

The primary endpoint is feasibility of the PostStroke-Manager with digital and personal support in a typical outpatient setting, starting very early after the qualifying event and thus during hospital stay. To use patient-reported outcome measures for evaluation, a questionnaire was designed that covers individual benefits regarding the digital system on the whole and the personal assistance provided by the stroke pilot (two questions), the digital components of the system except for the psychometric assessment (9 questions) and the technical support (3 questions related to the FAQ database, the ticket system and the contact with the stroke pilot). Patients will answer these questions at the end of the follow-up period, using a 6-point scale with the following options: “Very high benefit”, “high benefit”, “sufficient benefit”, “some benefit”, “rare benefit” and “no benefit”. The PostStroke-Manager concept will be judged feasible when at least 50% of the above 14 questions are rated as positive by at least 75% of patients. Here, the criterion “positive” corresponds to the answers “very high benefit”, “high benefit”, “sufficient benefit”, and “some benefit”. Furthermore, patients will be asked about their opinion regarding the assistance favored in the future, i.e., whether assistance should be provided by a digital system (app), or stroke pilots, or both (5-point scale: “surely pilots”, “rather pilots”, “both in a similar manner”, “rather app”, “surely app”).

Secondary endpoints include feasibility with regard to the three groups TIA, ischemic and hemorrhagic stroke. Furthermore, qualitative feedback from participating stroke pilots and primary care physicians on the feasibility of implementing the PostStroke-Manager concept will help to gain insight into the perspective of health care providers in addition to the patients’ perspective. Descriptive analyses are planned on secondary cerebrovascular events, the 1-year course of psychometric assessments (e.g., HADS, SF-36), laboratory, clinical variables and stroke-related impairments (NIHSS, mRS, BI) as well as blood pressure measurements, ECG signals, physical activity and medication adherence as recorded digitally by an automatically generated daily question and standardized questionnaires (AAQ, ABQ). Parameters will be evaluated in the overall sample as well as regarding the three groups of diagnoses. To consider economic aspects, days of hospitalization, details on rehabilitations and the frequency of medical services (e.g., physical therapies, consultations of the primary care physician) will be assessed. In addition to these analyses, technical problems will be recorded and signals originating from the activity tracker (including accelerometer and gyroscope) and the blood pressure monitor will be used for biomedical interpretations.

### Sample size calculation

Due to the observational nature of the study and the primary endpoint, as assessed by a questionnaire presented to all patients, typical sample size calculations focusing on group comparisons are not applicable. For secondary endpoints, such as feasibility regarding the three included diagnoses, an estimated sample size calculation was performed with an effect size ranging between of 0.35 and 0.65, an α-error of 0.05 and a power (1-β-error) of 0.85 using the statistical toolbox “G*Power” (version 3.1.9.6 [39];). This calculation revealed that statistically significant differences between the groups TIA, ischemic and hemorrhagic stroke, can be found when using a sample size of 30 patients each group, resulting in an overall sample size of 90 patients.

### Contacts

This study is part of a joint project between the Department of Neurology and Innovation Center Computer Assisted Surgery (ICCAS), both affiliated with Leipzig University, Leipzig, Germany. The medical content of the study has been designed and will be conducted by the Department of Neurology at the University of Leipzig, while the technical environment has been implemented and will be managed by ICCAS. Within the study, contractual arrangements are used to implement collaboration with primary care physicians, including study- and patient-related communication and data entry into the system. Additional contractual arrangements will be used for the collaboration with rehabilitation facilities. The evaluation of the study will be performed by the PostStroke-Manager consortium.

## Perspective

By combining a digital health care app that includes 10 components addressing the complex requirements in the setting of post stroke management, and intensive personal assistance by specially trained stroke pilots, the PostStroke-Manager concept represents a novel approach for patient-centered care.

This comprehensive approach integrates new developments in the field of medical care, personal interactions, mobile devices, data handling, and protection, all applied in a typical outpatient setting. Although all mobile devices fulfill the standard of an already existing CE certification, their uncontrolled use together with a newly created app in an outpatient setting, which would directly impact on medical decisions, is excluded due to regulatory requirements. Therefore, this study aims to demonstrate feasibility of such an approach as a pre-condition for further developments. In this light, the primary endpoint was intentionally chosen to not include efficacy elements such as a reduction of secondary vascular events. Instead, patient-reported outcomes [40] are used to capture the patients’ perspective on the combined digital and personal support. To obtain valid information on this topic, this study will include patients capable of using an app and mobile devices. In case of a positive evaluation of the PostStroke-Manager concept, efficacy and the usability by a broader spectrum of patients will need to be evaluated in a subsequent study. Methodologically, this approach will require the inclusion of a control group to allow comparison with usual care, a larger number of patients, with statistical power calculations that can build on the present study, and a follow-up period of several years to allow realistic detection of secondary events. This subsequent study should also include a group of patients with limited ability to use mobile devices and must grant access to caregivers. This will help to provide insight into the extent to which family and caregiver support is needed in the use of digital technologies.

To address different needs and priorities that may arise in secondary stroke prevention with reference to the first cerebrovascular event, this feasibility study includes patients with ischemic stroke,TIA, and cerebral hemorrhage. For the planned secondary endpoints, this stratification may help to figure out which patients may experience the combined digital and personal support as most beneficial.

More generally, this study will provide information on the critical patient-centered perspective regarding digital-based supports, which will ultimately help to improve conceptual aspects of future digital applications. Further, this study will generate insights into the potential use of mobile devices and digital technology for remote care as an option that has recently gained importance to maintain stroke care in situations with limited face-to-face contacts [41, 42].

## Data Availability

The dataset generated and analyzed during the study is not publicly available due to the included personal data of patients. For scientific analyses, pseudonymized and anonymized data will be used by the PostStroke-Manager consortium. Generally, data will be stored for at least 10 years. Anonymized data may also be used for meta-analyses upon reasonable request according to the recommendation of the International Committee of Medical Journal Editors (ICMJE).

## Abbreviations

AAQ: Adherence assessment questionnaire
ABQ: Adherence barriers questionnaire
App: Application
BI: Barthel index
BT: Bells test
DRKS: German Register for Clinical Trials
ECG: electrocardiography
EQ-5D: EuroQoL 5 dimensions
FAQs: Frequently asked questions
HADS: Hospital anxiety and depression scale
HCEI: Healthcare empowerment inventory
ICCAS: Innovation Center Computer Assisted Surgery
MMSE: Mini mental status examination
mRS: modified Rankin scale
NIHSS: National institutes of health stroke Scale
PHQ: Patient health questionnaire 9 items
pre-mRS: modified Rankin scale before the event
QR: Quick response
SF36: Short form (36) health survey
TMT: Trail making test
